# Dietary Supplementation With Olive Leaf Extract Alleviated Lipid Accumulation and Improved Growth and Intestinal Flora in *Trachinotus ovatus* Fed High‐Fat Diet

**DOI:** 10.1155/anu/8894218

**Published:** 2026-08-21

**Authors:** Jiaying Xie, Jiajian Shen, Douglas R. Tocher, Yuanyou Li, Yansen Hao, Zeling Lin, Han Zhan, Fan Lin, Shuqi Wang, Cuiying Chen

**Affiliations:** ^1^ Guangdong Provincial Key Laboratory of Marine Biotechnology, Institute of Marine Sciences, Shantou University, Shantou 515063, China, stu.edu.cn; ^2^ College of Marine Sciences and Guangdong Laboratory for Lingnan Modern Agriculture, South China Agricultural University, Guangzhou 510642, China, scau.edu.cn

**Keywords:** HFD, intestinal health, lipid accumulation, OLE, *Trachinotus ovatus*

## Abstract

With the application of high‐fat diets (HFDs) leading to increased prevalence of fatty liver in aquaculture, plant extracts such as olive (*Olea Europaea L.*) leaf extract (OLE) have attracted much attention due to their potential bioactive effects. In the present study, five experimental groups were established with HFD (15.5% crude lipid) supplemented with 0.0%, 0.5%, 1.0%, 1.5%, and 2.0% OLE, respectively, and each was fed to three replicate cages containing 20 juvenile *Trachinotus ovatus* (initial body weight: 11.57 ± 0.19 g) per cage. Fish fed HFD supplemented with 1.0% OLE showed significantly increased growth and survival and reduced lipid content in whole fish and triglyceride (TG) and cholesterol contents in serum compared to fish fed HFD (*p* < 0.05). Analysis of liver gene expression showed that reduced lipid content might be associated with reduced expression of genes associated with lipid synthesis (sterol regulatory element binding protein 1 [*srebp1*], fatty acid synthase [*fas*], acetyl‐CoA carboxylase [*acc*], and diacylgycerol acyltransferase 1 [*dgat1*]), and increased expression of genes associated with fatty acid oxidation (peroxisome proliferator‐activated receptor α [*pparα*], hormone‐sensitive lipase [*hsl*], and carnitine palmitoyl transferase‐1 [*cpt1*]) and lipid transport (CD36 molecule [*cd36*] and fatty acid binding protein 1 [*fabp1*]). Inclusion of OLE not only significantly lowered serum aspartate aminotransferase (AST) and alanine aminotransferase (ALT) activities but also increased activities of serum alkaline phosphatase (AKP) and superoxide dismutase (SOD) and total antioxidant capacity (TAOC), while serum malondialdehyde (MDA) decreased in fish fed HFD with 0.5%–1.0% OLE compared to fish fed HFD (*p* < 0.05). Additionally, 0.5%–1.0% dietary OLE significantly enhanced the integrity of intestinal morphology, reduced intestinal lipase activity and expression of inflammatory factors (interleukin‐1 [*il-1*] and *il-8*), and increased the diversity of intestinal microbiota (*p* < 0.05). The abundance of beneficial bacterial taxa (*Bacteroides*) was increased, while harmful bacterial taxa (*Mycoplasma*) decreased in *T. ovatus* fed diets with OLE compared to fish fed HFD. The data suggested that dietary OLE (particularly 1.0%) alleviated the negative effects of HFD in *T. ovatus*, which has highlighted the positive impacts of dietary OLE in fish and provided a new development opportunity for the olive industry through utilization of production waste.

## 1. Introduction

Fishmeal is considered an ideal ingredient in aquatic feed because of its high protein content, essential amino acid composition, and easy digestion [1]. However, due to finite and limited supply, the price of fishmeal has increased, which, in turn, has led to a rise in the cost of feed and aquaculture production [2]. Lipids (oils and fats) are the most effective ingredients for achieving the best balance between protein and energy contents in aquatic feeds [3]. In fish, an appropriate dietary lipid level can provide high energy density, improve the absorption of nutrients, and enhance feed conversion [4, 5]. Therefore, high‐fat diets (HFDs) are capable of effectively supplying adequate energy in feed and reserving protein for growth. In this way, HFD can enhance feeding efficiency and cut down farming expenses [6]. However, excessive fat intake in fish can lead to adverse effects, including increased lipid deposition in tissues and metabolic disorders, which may induce related diseases and, consequently, result in economic losses. For example, feeding HFD increased lipid droplet content and damaged the morphological structure of the hepatopancreas and reduced antioxidant capacity in tilapia (*Oreochromis niloticus*) [7]. Other studies in teleosts showed that excess dietary lipid can disturb the oxidative status and metabolic enzyme activities in largemouth bass (*Micropterus salmoides*) [4], while HFD was associated with increased lipid deposition and intestinal damage in juvenile *Monopterus albus* [8]. Therefore, identifying safe and effective ways to improve the utilization of feed while alleviating the negative effects of the use of HFD is an urgent issue in the field of aquatic animal nutrition.

Recently, there has been growing interest in feed supplements with the potential to reduce the risk of disorders of metabolism and to mitigate the development of fatty liver in cultured fish fed HFD. Olive (*Olea Europaea L*.) leaf extract (OLE) is a phytogenic feed additive containing a variety of bioactive phenolic compounds, including oleuropein, hydroxytyrosol, luteolin, and rutin, among others [9]. Previous studies indicated that the bioactives in OLE can exert a variety of physiological effects in both humans and animals [10], including antioxidant, anti‐inflammatory, antihypertensive, and metabolic regulatory activities, with potential benefits for obesity‐related metabolic disturbances and cardiometabolic risk factors [11–13]. Given the known functional properties of OLE bioactives in mammals, there has been increasing interest in the application of OLE in aquafeeds. It was shown that dietary inclusion of OLE or olive leaf powder can enhance the growth, survival rate, immunity, and health of fish [14]. A study conducted on common carp (*Cyprinus carpio*) also reported that the carcass lipid content was significantly reduced in fish fed a standard diet supplemented with 200 mg/kg OLE [15]. However, the specific mechanisms of OLE actions in mitigating metabolic disorders and hepatic lipid deposition induced by HFD in fish are still unclear.

Golden pompano (*Trachinotus ovatus*) is a carnivorous marine fish that can thrive on formulated feeds and is characterized by rapid growth, strong resistance to adverse conditions, and high survival rates in aquaculture conditions. These traits have made it an economically important fish species with high suitability for cage culture in the marine aquaculture industry in China. However, it is important to highlight a currently widespread phenomenon, which is that deep‐sea intensive culture and the long‐term use of HFDs likely cause oxidative and inflammatory stress, resulting in fatty liver and growth suppression in fish [16, 17]. Thus, it is of increasing importance to find functional feed additives, optimize formulations, and develop high‐efficiency, low‐cost, and environmentally friendly compound feeds for the cultivation of *T. ovatus*. Previous studies have shown that 42%–49% protein and 6.5%–12.5% lipid are suitable dietary ranges for the growth and health of *T. ovatus* [18]. When the dietary lipid level was increased to more than 14%, growth and feed intake of juvenile *T. ovatus* decreased, and lipid content of the liver increased and triggered oxidative stress [19–21]. In the present study, we utilized a dietary lipid level of 16% to establish an HFD model. Hence, the current study aimed to elucidate the impacts of dietary OLE (~40% oleuropein) supplementation on the negative effects induced by feeding HFD in *T. ovatus*, including growth performance suppression, lipid metabolism disorders, inflammation, oxidative damage, and intestinal health problems in *T. ovatus*. The results provide a basis for optimizing compound feed suitable for the growth of *T. ovatus* and suggest that utilization of production waste could be a new development opportunity for the olive industry.

## 2. Materials and Methods

### 2.1. Animal Ethics Statement

All experimental procedures involving live fish were carried out in full accordance with the Guide for the Care and Use of Laboratory Animals of the National Institutes of Health (NIH Publications, Number 8023, revised in 1978). In addition, all animal experimentation was reviewed and approved by the Shantou University Animal Care and Use Committee (Number 132, revised on 6 July 2022).

### 2.2. Diets

A set of five diets was formulated to provide ~42% crude protein with fishmeal and soy protein concentrate (SPC) as the predominant sources of protein and around 16% lipid using mixed vegetable oil (palm oil/rapeseed oil/perilla oil, 3:1:1 by vol.) and soybean lecithin as the major added fat sources. On this basis, diets were designed with graded levels of OLE of 0.0%, 0.5%, 1.0%, 1.5%, and 2.0%. The commercial OLE product contained 40.3% oleuropein, 9.3% hydroxytyrosol, 4.2% apigenin, 3.5% luteolin, 1.6% oleanolic acid, 1.1% maslinic acid, 0.5% chlorogenic acid, 0.6% caffeic acid, and around 3.0% ash and 35.0% fibrous organic substances insoluble in water and alcohol and was obtained from Yaan Huijie Biotechnology Co., Ltd. (Yaan city, China). The dietary ingredients and analyzed proximate compositions are shown in Table 1.

**Table 1:** Ingredients and proximate compositions of the experimental diets (% dry weight).

Ingredient (g/100 g diet)	Dietary OLE^a^ level (%)				
	0.0	0.5	1.0	1.5	2.0
Fishmeal	30.0	30.0	30.0	30.0	30.0
Soy protein concentrate	18.0	18.0	18.0	18.0	18.0
Corn gluten	10.0	10.0	10.0	10.0	10.0
Fermented soybean meal	10.0	10.0	10.0	10.0	10.0
Cassava starch	6.0	6.0	6.0	6.0	6.0
α‐Starch (corn starch)	5.0	5.0	5.0	5.0	5.0
Mixed vegetable oil^b^	10.0	10.0	10.0	10.0	10.0
Soybean lecithin	2.0	2.0	2.0	2.0	2.0
Carboxymethyl cellulose	3.0	2.5	2.0	1.5	1.0
Vitamin premix^c^	2.0	2.0	2.0	2.0	2.0
Mineral premix^d^	2.0	2.0	2.0	2.0	2.0
Lutein	0.2	0.2	0.2	0.2	0.2
Betaine	0.5	0.5	0.5	0.5	0.5
Choline chloride	0.5	0.5	0.5	0.5	0.5
Calcium dihydrogen phosphate	0.8	0.8	0.8	0.8	0.8
Olive leaf extract (OLE)	0.0	0.5	1.0	1.5	2.0
Total	100.0	100.0	100.0	100.0	100.0
Proximate composition (% dry weight)					
Dry matter	89.87	90.21	90.04	90.02	90.27
Organic matter	79.46	79.55	79.38	80.18	80.14
Crude protein	41.79	41.81	41.86	41.88	41.75
Crude fat	15.34	15.33	15.93	15.49	15.62
Crude fiber	3.07	3.18	3.06	2.93	2.81

^a^OLE: Olive leaf extract purchased from Chengdu Huijie Biotechnology Co., Ltd. contains 40.3% oleuropein, 9.3% hydroxytrosol, 3.5% luteolin, 4.2% apigenin, 1.6% oleanolic acid, 1.1% maslinic acid, 0.5% chlorogenic acid, 0.6% caffeic acid, and about 3.0% ash and 35% fibrous organic substances insoluble in water and alcohol.

^b^Mixed vegetable oil: palm oil/rapeseed oil/perilla oil (3:1:1, by vol).

^c^Vitamin premix (/kg)：≥150,000 IU vitamin A, ≤300,000 IU vitamin D_3_, ≥620 IU vitamin E, ≥200 mg vitamin K_3_, ≥500 mg vitamin B_1_, ≥500 mg vitamin B_2_, ≥1500 mg calcium pantothenate, ≥1500 mg nicotinamide, ≥80 mg folic acid, ≥500 mg vitamin B_6_, and carrier alpha‐cellulose.

^d^Mineral premix (/kg)：≤2000 mg KI, ≤200 mg CoCl_2_, ≤2500 mg CuSO_4_, ≤15,000 mg ZnSO_4_, ≤10,000 mg MnSO_4_, ≤2500 mg FeSO_4_, ≤50 mg Na_2_SeO_3_, and carrier alpha‐cellulose.

For the preparation of each diet, the dry ingredients were ground and thoroughly mixed before oil and water were incorporated to form a paste, which was extruded into appropriately sized pellets utilizing a laboratory pelleting machine (SLC‐45, Fishery Machinery and Instrument Research Institute, Shanghai, China). The pellets were air‐dried, sealed in plastic bags, and stored at −20°C until utilization.

### 2.3. Experimental Fish and Feeding Trial Design

The nutritional trial was conducted from the beginning of September to the end of November 2022 in floating sea cages ~150 m offshore of Nan Ao Marine Biology Station (NAMBS) of Shantou University. Juvenile *T. ovatus*, sourced from a fish hatchery in Zhangzhou, Fujian Province, were placed in a cuboid floating net cage (6.0 m × 6.0 m × 3.0 m) for 2 weeks prior to the commencement of the feeding trial. During this period, the fish were fed a diet containing a mixture of equal amounts of the five experimental feeds. By 1 week prior to the commencement of the feeding trial, the *T. ovatus* were acclimated to the experimental conditions such that there were no mortalities, and the fish were eating normally and were in good condition. The *T. ovatus* were starved for 24 h and anesthetized with 0.01% phenoxyethanol, and 300 healthy fish (average weight, 11.57 ± 0.10 g) were distributed randomly into 15 net sea cages (1.0 m × 1.0 m × 1.5 m) at a stocking density of 20 fish per cage. The experimental diets were assigned at random to three cages, and the fish were fed by hand to satiation twice daily (6:00 and 16:00) for 8 weeks, with the quantity of feed administered to each cage recorded each day. During the trial, the temperature of the seawater varied naturally with season between 20.0 and 29.6°C, while salinity levels ranged from 30 to 32‰, dissolved oxygen levels were in excess of 5.0 mg/L, and ammonia nitrogen levels were maintained below 0.05 mg/L.

### 2.4. Sample Collection

At the termination of the trial, all fish in each cage were starved for 24 h, anesthetized with 0.01% phenoxyethanol, counted, and weighed individually. Three fish were selected randomly from each cage and frozen at −20°C prior to the analysis of whole‐body composition. Samples of blood from three additional fish per cage were drawn from the caudal vein, and serum was obtained by centrifugation at 1500 × *g* for 10 min at 4°C for subsequent analysis of biochemical indicators. The viscera collected from the same three fish were weighted for the determination of viscerosomatic indices (VSIs). Then the liver was removed for these viscera and weighted to calculate the hepatosomatic indices (HSIs). Two portions of liver, intestine, and muscle were collected from each tissue sample for biochemical and enzyme activity analyses. Additionally, another sample from each liver and intestine was collected into cryotubes for molecular (gene expression) analyses. All the above tissue samples were frozen immediately in liquid nitrogen and stored at −80°C prior to the analyses. The samples of tissue from the three fish per cage were individually analyzed and then averaged to provide data per cage (*n* = 3). The liver and anterior intestine samples for histological analysis were collected from another three fish per cage and placed in a 4% solution of paraformaldehyde. In addition, on the day prior to the main sampling and before feed was withdrawn, intestinal contents were collected from four or six fish per cage and pooled in two pools of two or three per cage (*n* = 6) for microbiota analysis.

### 2.5. Growth Performance Indices

Growth indices were calculated as follows:

Weight gain WG,%=100×final body weight−initial body weight×initial body weight−1;

Specific growth rate SGR,%/d=100×lnfinal body weight−lninitial body weight×days of feeding trial−1;

Feed conversion ratio FCR=total dry weight of feed fed×final body weight−initial body weight−1;

Survival rate SR,%=100×final fish number×initial fish number−1;

Hepatosomatic index HIS,%=100×liver weight×fish body weight−1;

Viscerosomatic index VSI,%=100×visceral weight×fish body weight−1;

Condition factor CF, gcm−3=100×fish body weight, g×fish body length, cm−3.

### 2.6. Proximate Composition Analysis

Proximate compositions (moisture, protein, lipid, fiber, and ash) of the experimental diets and whole fish body (moisture, protein, lipid, fiber, and ash) were determined following standard methods. Specifically, moisture was assessed by drying the samples at 105°C until constant weight (AOAC, 2006). Protein contents were measured by determining nitrogen (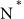
6.25) using a Kjeldahl System (Kjeltec8400; FOSS) according to the Chinese national standard method (GB/T 6432‐2006). Lipid contents were determined using an automatic Soxhlet extraction apparatus (SZF‐06A; Xinjia, Shanghai, China) with petroleum (boiling range 40–60°C) extraction for 4 h at 60–65°C according to the Chinese national standard method (GB/T 6433‐2006). Ash contents were obtained by incinerating the samples in a muffle furnace (CWF1100; Carbolite) at 550°C for 6 h based on the Chinese national standard method (GB/T 6438‐2007). Fiber content was analyzed using a fully automatic fiber analyzer (F2000; Hanon, China) according to the Chinese national standard method (GB/T6434‐2006). Total organic matter contents of feeds were obtained by calculation, subtracting ash contents from dry matter contents.

### 2.7. Total Lipid and Fatty Acid Composition Analysis

Liver fatty acid compositions were determined using a Shimadzu GC‐17A gas chromatograph (Kyoto, Japan). In brief, total lipids were isolated from the liver tissue using chloroform/methanol (2:1, by volume). Subsequent methylation was conducted with boron trifluoride etherate (concentration, 48%; Acros Organics, NJ, USA) to generate fatty acid methyl esters, following the analytical procedures detailed in a previous study [22]. Likewise, the specific gas chromatography settings and experimental protocols followed those outlined previously [23]. Fatty acids were identified and measured using established commercial reference standards (Sigma–Aldrich, St. Louis, MO, USA) and the Shimadzu CLASS‐GC10 gas chromatography workstation.

### 2.8. Serum Biochemical Parameters and Enzyme Activities Analysis

Serum triglyceride (TG, A110‐1‐1; 500 nm), total cholesterol (TC, A111‐1‐1; 500 nm), high‐density lipoprotein cholesterol (HDLC, A112‐1‐1; 550 nm), and low‐density lipoprotein cholesterol (LDLC, A113‐1‐1; 550 nm), along with serum antioxidant status parameters including malondialdehyde (MDA, A003‐1‐1; 532 nm), aspartate aminotransferase (AST, C010‐2‐1; 505 nm), alanine aminotransferase (ALT, C009‐2‐1; 505 nm), alkaline phosphatase (AKP, A059‐2; 520 nm), superoxide dismutase (SOD, A001‐3‐2; 450 nm), and total antioxidant capacity (TAOC, A015‐2‐1; 405 nm), were all measured using commercial assay kits (Jiancheng Biotech Co., China) that employed spectrophotometric assays (absorbance wavelengths as indicated). All assays were conducted in detail according to the manufacturer’s instructions, as described previously [20, 24].

### 2.9. Liver and Intestine Histological Analysis

Samples of both liver and intestine were preserved in a 4% solution of paraformaldehyde for fixation, and the acquisition and scanning of slices were carried out commercially (Wuhan Servicebio Technology Co., Ltd., Wuhan, China). Briefly, liver specimens were frozen and sectioned into 5 μm slices and subsequently stained with oil red O (ORO) following standard procedures as detailed previously [25]. In contrast, intestinal samples underwent dehydration and embedding in paraffin wax, and sections of 5 μm thickness were produced by a paraffin slicing machine (PM‐24, Servicebio, China) before being stained with hematoxylin and eosin (H&E). Histological images obtained were captured and analyzed utilizing CaseViewer software (SWE‐CX63, Servicebio). Lipid droplets stained by ORO were quantified using Image‐Pro Plus Version 6.0 (Media Cybernetics, Rockville, MD, USA).

### 2.10. Intestinal Enzyme Activities Analysis

The activities of amylase, trypsin, and lipase in the intestine were measured using commercial assay kits according to the manufacturer’s instructions (Nanjing Jiancheng Bioengineering Institute). Briefly, intestinal samples (three fish per replicate cage) were individually weighed and mechanically homogenized at a ratio of 0.1 g tissue to 0.9 mL in phosphate‐buffered saline (PBS) at 4°C, and the homogenates were centrifuged at 3500 × *g* for 10 min at 4°C to obtain supernatants for the assay. Samples of the supernatant were combined with starch and an iodine solution, and a blue complex was produced after hydrolysis of starch and then measured at 660 nm to determine amylase activity. A red product was generated after reacting supernatants with 6‐methyl resorufin‐labeled lipid and then measured at 580 nm to determine lipase activity. The activity of trypsin was calculated by detecting the change in absorbance at 253 nm after reacting supernatants with the arginine ethyl ester substrate. Protein contents of the supernatants were determined by a commercial kit (BCA Protein Assay Kit, G3522, GBCBIO Technologies Inc.). All enzyme activities were presented as units per milligram of protein (U/mg prot).

### 2.11. Intestinal Microbiome Analysis

DNA was extracted from the intestinal samples utilizing the MagicPure Stool and Soil Genomic DNA Kit (Tiangen, Beijing, China), following the manufacturer’s prescribed protocol. The quantity and quality of the extracted DNA were assessed using the Qubit dsDNA HS Assay Kit and Qubit 3.0 Fluorometer (Invitrogen, Thermo Fisher Scientific, Oregon, USA). Subsequent to standardization at 1 ng/μL, equal volumes of DNA samples from three fish in each replicate were pooled across treatments, resulting in six composite samples per dietary group. These pooled samples underwent microbial community profiling via high‐throughput sequencing, outsourced to Biomarker Technologies Co., Ltd. (Beijing, China). Once sequences were optimized, they underwent operational taxonomic unit (OTU) clustering analysis and species classification annotation. Analysis of the diversity index was performed based on OTU clustering, and in‐depth examination of species structure and differential species analysis was conducted using the available taxonomic information [26]. All the above bioinformatic analyses were carried out using BMK Cloud (Biomarker Technologies Co., Ltd.).

### 2.12. RNA Extraction, cDNA Synthesis, and Quantitative Real‐Time PCR

Total RNA from liver and intestine samples was extracted using the Trizol reagent (Vazyme, China) following the manufacturer’s instructions, and the concentration and purity of the extracted RNA were quantified using a NanoDrop One Spectrophotometer (Thermo Fisher Scientific, USA). Only samples exhibiting an A260/A280 ratio between 1.8 and 2.2, with a total yield exceeding 5 μg, were utilized. The integrity of RNA was further validated via 1.0% agarose gel electrophoresis. Subsequently, cDNA synthesis from high‐quality RNA was performed using HiScript II Q Select RT SuperMix for gPCR kits (Vazyme, China), following the manufacturer’s instructions. Transcript levels of genes of lipid metabolism and inflammatory responses were determined by SYBR Green‐based real‐time quantitative PCR (qPCR) using primers, as shown in Table S1. All qPCRs were carried out in a LightCycle 480 Real‐Time PCR System (Roche, Switzerland) using the ChamQ Universal SYBR qPCR Master Mix kit (Vazyme, China). The reaction systems were filled with 3 μL of nuclease‐free water, 5 μL of SYBR Green Supermix, 0.5 μL each of forward and reverse primers, and 1 μL of cDNA. The thermal cycling conditions were initial denaturation at 94°C for 5 min; 45 cycles of denaturation at 95°C for 10 s, annealing at 58°C for 20 s, and extension at 72°C for 20 s, followed by a final dissociation step at 95°C for 5 s, 65°C for 1 min, and cooling at 40°C for 10 s. Detailed operational steps and experimental conditions have been published previously [20]. Relative mRNA levels of target genes were normalized with *β-actin* expression and calculated by the 2^−ΔΔCT^ method, as described in detail previously [27].

### 2.13. Calculation and Statistical Analysis

All data were analyzed statistically using the general linear mixed model in SPSS Statistics Version 20.0 software (SPSS Inc., Chicago, IL, USA) as follows:

Y=Xβ+Zγ+ε,

where *Y* is the observed dependent variable; *X* is the design matrix for fixed effects (constructed from user‐specified predictors like dietary OLE level); *β* is a vector of fixed‐effect coefficients (parameters estimated, representing the average effects of predictors); *Z* is the design matrix for random effects (links observations to the groups/individuals from which random effects are drawn); *γ* is a vector of random‐effect coefficients; and *ε* is a vector of residual errors.

Assessments of the linear and quadratic effects of dietary OLE levels were carried out by orthogonal polynomial analysis. Significant differences among groups were determined by one‐way analysis of variance (ANOVA) using Tukey’s honest significant difference (HSD) test. Differences between means with a statistical *p* value of <0.05 were considered significant.

## 3. Results

### 3.1. Dietary Supplementation With OLE Improved Growth Performance and Reduced Lipid Accumulation in *T. ovatus* Fed HFD

After the 8‐week feeding trial, there were significant impacts on final weight, WG, SGR, and SR as dietary OLE levels increased from 0% to 2.0% (Table 2). Final weight, WG, and SGR showed significant quadratic responses (*p* < 0.05) being significantly increased and then decreased with increasing levels of OLE in the diet, with 1.0% OLE shown to be the optimal concentration. Similarly, SR increased with increasing dietary OLE up to 1.0%, and then decreased as the level of dietary OLE increased further, displaying a significant quadratic response (*p* < 0.05). Although FCR, CF, HSI, and VSI all showed no significant differences with OLE supplementation, feed efficiency showed an improving trend up to 1.0%, while HSI showed a decreasing trend with dietary OLE supplementation.

**Table 2:** Growth performance, feed utilization, and biological indices of *Trachinotus ovatus* fed high‐fat diets with different levels of olive leaf extract (OLE) for 8 weeks^1^.

Parameters	Dietary OLE level (%)					Pooled SEM	*p* value		
	0.0	0.5	1.0	1.5	2.0		ANOVA	Linear	Quadratic
Initial weight (g)	11.48	11.59	11.71	11.54	11.54	0.126	0.732	0.890	0.288
Final weight (g)	60.81^bc^	68.20^b^	79.57^a^	57.74^c^	58.65^c^	1.844	<0.001	0.030	<0.001
WG (%)	429.56^bc^	488.50^b^	579.35^a^	400.48^c^	408.79^c^	15.807	<0.001	0.027	<0.001
SGR (% day^−1^)	2.98^bc^	3.16^b^	3.42^a^	2.87^c^	2.90^c^	0.050	<0.001	0.019	<0.001
FCR	1.91	1.79	1.68	1.73	1.80	0.063	0.214	0.211	0.050
SR (%)	68.33^b^	85.00^a^	86.67^a^	78.33^ab^	80.00^ab^	3.162	0.016	0.127	0.007
HSI (%)	2.39	2.01	2.11	2.06	2.00	0.190	0.195	0.515	0.424
VSI (%)	8.67	9.15	8.91	8.38	8.65	0.390	0.268	0.185	0.164
CF (gcm^−3^)	3.31	3.17	3.12	3.12	3.28	0.237	0.961	0.477	0.924

*Note:* Means in the same row with different superscript letters are significantly different at *p* < 0.05 as determined by one‐way ANOVA. The linear and quadratic effects of OLE levels were assessed using orthogonal polynomial contrasts with a significance threshold of *p*  < 0.05.

Abbreviations: CF, condition factor; FCR, feed conversion ratio; HSI, hepatosomatic index; SGR, specific growth rate; SR, survival rate; VSI, viscerosomatic index; WG, weight gain.

^1^Values are the means of three replicates.

**Figure 1:**
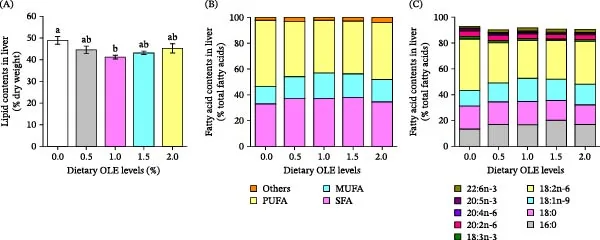
Lipid content and fatty acid composition of liver of *Trachinotus ovatus* fed high‐fat diets with different levels of olive leaf extract (OLE) for 8 weeks. (A) Lipid content of liver (% dry weight). (B) Composition (percentage of total fatty acids) of saturated fatty acids (SFAs), monounsaturated fatty acids (MUFAs), polyunsaturated fatty acids (PUFAs), and others (peaks of unknown identity) in liver. (C) The principle individual fatty acids (percentage of total fatty acids) in liver. Bars with different superscript lowercase letters in Figure 1A are significantly different (*p* < 0.05) as determined by one‐way ANOVA.

**Figure 2:**
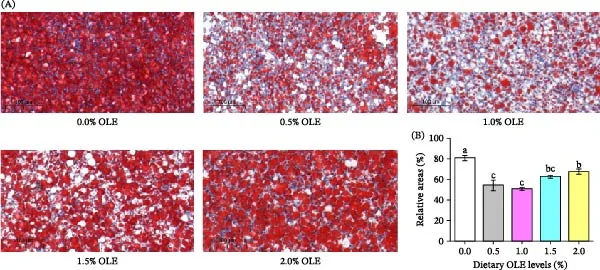
Histological examination of liver of *Trachinotus ovatus* fed high‐fat diets with different levels of olive leaf extract (OLE) for 8 weeks. (A) Sections of liver stained with oil red O (scale bar, 100 μm). (B) Quantitation of the relative area (percentage) of lipid droplets (stained red) in hepatic sections. Values are presented as means ± SEM (*n* = 3). Bars with different superscript lowercase letters denote significant differences (*p* < 0.05) as determined by one‐way ANOVA.

The lipid content of the whole body decreased and then increased with increasing dietary OLE, with 1.0% OLE showing the lowest lipid content, whereas 1.5% OLE did not show any reduction in whole‐body lipid content (Table 3). Levels of TG and TC in serum decreased significantly as dietary OLE supplementation level increased up to 1.0% and then decreased with a further increased level of OLE with obvious linear and quadratic trends (*p* < 0.05) (Table 4). The levels of HDLC increased with 1.0% dietary OLE (*p* < 0.05), and then decreased with the further increased OLE addition, while LDLC showed the opposite trend. (Table 4). In addition, total lipid content of liver (% dry weight) also exhibited a significant downward trend as dietary OLE level increased to 1.0% (*p* < 0.05) (Figure 1A). Moreover, liver contents of total saturated fatty acids (SFAs) and monounsaturated fatty acids (MUFAs), especially 16:0, 18:0, and 18:1n‐9, showed trends of first increasing and then decreasing, with the critical point being 1.0% OLE (Figure 1B, C). The proportions of long‐chain polyunsaturated fatty acids (LC‐PUFAs), including 20:4n‐6, 20:5n‐3, and 22:6n‐3 showed similar trends (Table S2). However, the proportion of the total PUFA, especially 18:2n‐6, showed the opposite trend (Figure 1B C). ORO staining of liver sections showed that the range of lipid droplet size was the least and lipid droplets were significantly smaller, reduced in number, and distributed more evenly with dietary supplementation with OLE of 1.0% (*p* < 0.05) (Figure 2).

**Table 3:** Proximate compositions of whole body of *Trachinotus ovatus* fed high‐fat diets with different levels of olive leaf extract (OLE) for 8 weeks^1^.

Parameters	Dietary OLE level (%)					Pooled SEM	*p* value		
	0.0	0.5	1.0	1.5	2.0		ANOVA	Linear	Quadratic
Whole body									
Moisture	69.55	67.98	70.66	67.71	70.34	1.127	0.296	0.721	0.525
Protein	15.60	16.93	15.87	17.19	15.54	0.565	0.190	0.940	0.121
Lipid	10.72	10.68	9.13	10.80	10.17	0.378	0.052	0.433	0.179
Ash	3.30	4.09	3.33	3.57	3.20	0.238	0.135	0.359	0.174

*Note:* The linear and quadratic effects of OLE levels were assessed using orthogonal polynomial contrasts with a significance threshold of *p*  < 0.05.

^1^Values are the means of three replicates.

**Table 4:** Serum biochemical and antioxidant parameters of *Trachinotus ovatus* fed high‐fat diets with different levels of olive leaf extract (OLE) for 8 weeks^1^.

Parameters	Dietary OLE level (%)					Pooled SEM	*p* value		
	0.0	0.5	1.0	1.5	2.0		ANOVA	Linear	Quadratic
TG (mmol L^−1^)	2.87^a^	1.25^c^	1.12^c^	2.05^b^	2.09^b^	0.070	<0.001	0.007	<0.001
TC (mmol L^−1^)	4.56^a^	3.70^b^	2.31^d^	3.29^c^	3.14^c^	0.055	<0.001	<0.001	<0.001
HDLC (mmol L^−1^)	1.20^b^	1.47^ab^	1.70^a^	1.39^ab^	1.59^ab^	0.102	0.047	0.055	0.102
LDLC (mmol L^−1^)	1.14^a^	1.01^a^	0.47^b^	0.66^b^	0.65^b^	0.046	<0.001	<0.001	<0.001
AKP (U 100 mL^−1^)	2.32^c^	2.68^bc^	3.91^a^	3.16^b^	2.68^bc^	0.151	<0.001	0.030	<0.001
ALT (U L^−1^)	40.19^a^	21.95^b^	25.36^b^	37.69^a^	47.60^a^	2.329	<0.001	0.002	<0.001
AST (U L^−1^)	30.03^a^	23.37^b^	19.94^b^	22.63^b^	32.72^a^	1.417	<0.001	0.326	<0.001
MDA (nmol mL^−1^)	5.54^a^	2.34^c^	2.79^bc^	2.34^c^	4.10^ab^	0.349	<0.001	0.026	<0.001
SOD (U mL^−1^)	12.18^b^	15.16^ab^	17.08^a^	15.76^ab^	15.82^ab^	1.022	0.062	0.035	0.039
TAOC (U mL‐1)	23.35^b^	28.14^ab^	32.93^a^	29.87^ab^	26.85^ab^	1.642	0.022	0.124	0.003

*Note:* Means in the same row with different superscript letters are significantly different at *p* < 0.05 as determined by one‐way ANOVA. The linear and quadratic effects of OLE levels were assessed using orthogonal polynomial contrasts with a significance threshold of *p* < 0.05.

Abbreviations: AKP, alkaline phosphatase; ALT, alanine aminotransferase; AST, aspartate aminotransferase; HDLC, high‐density lipoprotein cholesterol; LDLC, low‐density lipoprotein cholesterol; MDA, malondialdehyde; SOD, superoxide dismutase; TAOC, total antioxidant capacity; TC, total cholesterol; TG, triglyceride.

^1^Values are the means of three replicates.

### 3.2. Dietary OLE Supplementation Reduced Hepatic Lipid Deposition by Inhibiting Lipid Synthesis and Promoting Lipid Catabolism and Transport in *T. ovatus* Fed HFD

**Figure 3:**
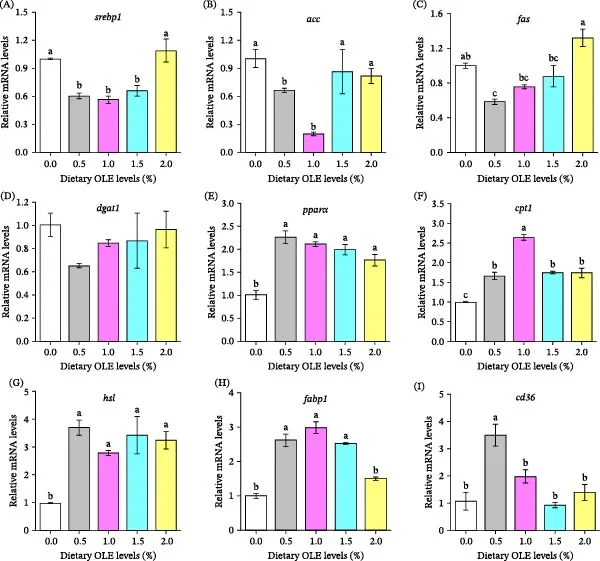
Relative expression of genes related to lipid metabolism in liver of *Trachinotus ovatus* fed high‐fat diets with different levels of olive leaf extract (OLE) for 8 weeks. (A–D) mRNA expression levels of genes related to lipid biosynthesis. (E–G) mRNA expression levels of genes related to lipid catabolism. (H, I) mRNA expression levels of genes related to lipid transport. Values are presented as means ± SEM, *n* = 3. Bars with different superscript lowercase letters are significantly different (*p* < 0.05) as determined by one‐way ANOVA. *acc*, acetyl‐CoA carboxylase; *cd36*, CD36 molecule; *cpt1*, carnitine palmitoyl transferase‐1; *dgat1*, diacylgycerol acyltransferase 1; *fabp1*, fatty acid binding protein 1; *fas*, fatty acid synthase; *hsl*, hormone‐sensitive lipase; *pparα*, peroxisome proliferator‐activated receptor α; *srebp1*, sterol regulatory element binding protein‐1; *β-actin*, actin beta.

Relative mRNA expression levels of genes related to lipid biosynthesis, including sterol regulatory element binding protein 1 (*srebp1*), acetyl‐CoA carboxylase (*acc*) and fatty acid synthase (*fas*), in liver decreased significantly when 0.5%–1.0% OLE was added to the diet compared to fish fed the control diet (0% OLE) (*p* < 0.05), but then increased significantly as dietary OLE level exceeded 1.0% (*p* < 0.05) (Figure 3A–C). The relative mRNA expression of diacylgycerol acyltransferase 1 (*dgat1*) also showed a similar trend, albeit not statistically significant (Figure 3D). Conversely, mRNA expression levels of genes of lipid catabolism, including peroxisome proliferator‐activated receptor α (*pparα*), carnitine palmitoyl transferase‐1 (*cpt1*), and hormone‐sensitive lipase (*hsl*), and lipid transport, including CD36 molecule (*cd36*) and fatty acid binding protein 1 (*fabp1*), increased significantly with dietary supplementation of OLE, particularly 0.5%–1.0%, compared with the control diet without OLE (*p* < 0.05) (Figure 3E–I).

### 3.3. Dietary OLE Supplementation Improved Liver Health and Immune and Antioxidant Capacities of *T. ovatus* Fed HFD

Serum activities of ALT and AST displayed a significant quadratic trend (*p* < 0.05). Liver health was improved by supplementation of 0.5%–1.0% OLE to the diet, as indicated by significantly lower activities of ALT and AST compared to fish fed the diet without OLE (*p* < 0.05) (Table 4). However, the activities of ALT and AST tended to rise as the dietary level of OLE increased further (Table 4). On the contrary, serum AKP activity showed a significant quadratic trend (*p* < 0.05) and increased significantly with dietary supplementation of OLE up to 1.0%, but tended to decrease as dietary OLE increased further. Regarding antioxidant capacity, SOD activity, TAOC, and MDA contents of serum all showed significant quadratic trends. Thus, SOD and TAOC increased as the level of dietary OLE increased to 1.0%, and then decreased as OLE supplementation increased further (*p* < 0.05), while MDA decreased up to 1.5% OLE before it increased at 2.0% OLE (*p* < 0.05) (Table 4).

### 3.4. Dietary OLE Supplementation Alleviated Intestinal Inflammation and Damage by Inhibiting the Expression of Inflammatory Factors in *T. ovatus* Fed HFD

**Figure 4:**
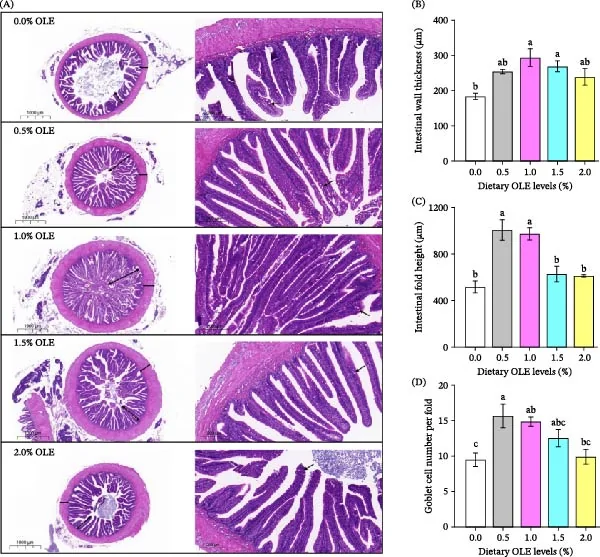
Morphology of the anterior intestine of *Trachinotus ovatus* fed high‐fat diets with different levels of olive leaf extract (OLE) for 8 weeks. (A) The entire section (left; scale bar, 1000 μm) and part section (right; scale bar, 200 μm) of the anterior gut of fish fed diets supplemented with 0.0%, 0.5%, 1.0%, 1.5% and 2.0% OLE, respectively. The location of the black lines, line segments with arrows at both ends and single ended arrows point to wall thickness, villus length and goblet cell, respectively. (B–D) The anterior gut morphology parameters of *Trachinotus ovatus*. Data shown as means ± SEM of three replicates. Bars in the same subfigure with different superscript lowercase letters are significantly different (*p* < 0.05) as determined by one‐way ANOVA.

**Figure 5:**
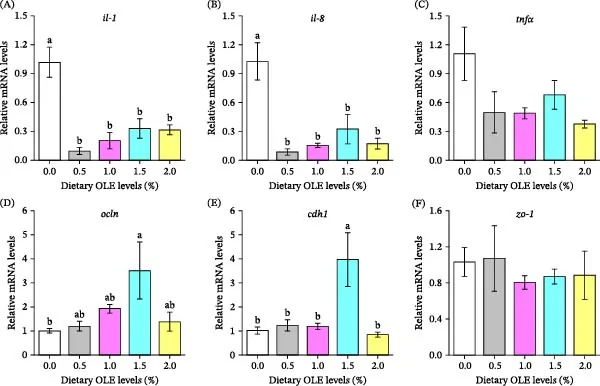
Relative expression of genes related to inflammatory responses and intestinal barrier function in intestine of *Trachinotus ovatus* fed high‐fat diets with different levels of olive leaf extract (OLE) for 8 weeks. (A–C) mRNA expression levels of genes related to inflammatory responses. (D–F) mRNA expression levels of genes related to intestinal barrier function. Values are presented as means ± SEM, *n* = 3. Bars with different superscript lowercase letters are significantly different (*p* < 0.05) as determined by one‐way ANOVA. *cdh1*, cadherin 1; *il-1*, interleukin‐1; *il-8*, interleukin‐8; *ocln*, occludin; *tnfα*, tumor necrosis factor alpha; *zo-1*, zonula occludens‐1; *β-actin*, actin beta.

Histological analysis of the intestine showed that dietary OLE improved the integrity and increased the length of intestinal microvilli of *T. ovatus* (Figure 4). Moreover, the addition of OLE to the diet reduced the mRNA expression levels of proinflammatory factors in the intestine, including interleukin‐1 (*il-1*) and *il-8* (*p* < 0.05) (Figure 5A, B). The expression of another proinflammatory factor, tumor necrosis factor *α* (*tnfα*), also showed a downward trend in the intestine of *T. ovatus* fed HFD diet supplemented with OLE (Figure 5C). Conversely, the mRNA expression levels of the intestinal epithelial barrier‐related genes, occludin (*ocln*) and cadherin 1 (*cdh1*), were upregulated in the intestine in fish fed diets including 1.0%–1.5% OLE (*p* < 0.05) (Figure 5D, E), while dietary OLE did not affect the expression of another intestinal epithelial barrier gene, zonula occludens protein 1 (*zo-1*) (Figure 5F).

### 3.5. Dietary OLE Supplementation Reduced the Absorption of Lipid by the Intestine and Altered the Composition of Intestinal Microbiota in *T. ovatus* Fed HFD

As shown in Table 5, the activity of lipase decreased in the intestine of *T. ovatus* fed HFD with 0.5%–1.5% OLE and then significantly increased with the addition of OLE up to 2.0% (*p* < 0.05). The addition of 0.5%–1.0% OLE to the HFD diet enhanced the activity of intestinal trypsin, but activity decreased significantly with the addition of more than 1.5% (*p* < 0.05). Dietary OLE did not affect amylase activity in the intestine of *T. ovatus* (*p* > 0.05).

**Table 5:** Activities (U/mg prot) of intestinal digestive of *Trachinotus ovatus* fed high‐fat diets with different levels of olive leaf extract (OLE) for 8 weeks^1^.

Parameters	Dietary OLE level (%)					Pooled SEM	*p* value		
	0.0	0.5	1.0	1.5	2.0		ANOVA	Linear	Quadratic
Lipase	2.51^ab^	1.47^bc^	1.49^bc^	1.30^b^	2.69^a^	0.227	0.003	0.799	<0.001
Amylase	0.14	0.13	0.18	0.15	0.09	0.031	0.473	0.433	0.189
Trypsin	621.78^ab^	649.66^ab^	683.08^a^	382.70^ab^	314.86^b^	78.757	0.023	0.005	0.105

*Note:* Means in the same row with different superscript letters are significantly different at *p* < 0.05 as determined by one‐way ANOVA. The linear and quadratic effects of OLE levels were assessed using orthogonal polynomial contrasts with a significance threshold of *p* < 0.05.

^1^Values are the means of three replicates.

**Figure 6:**
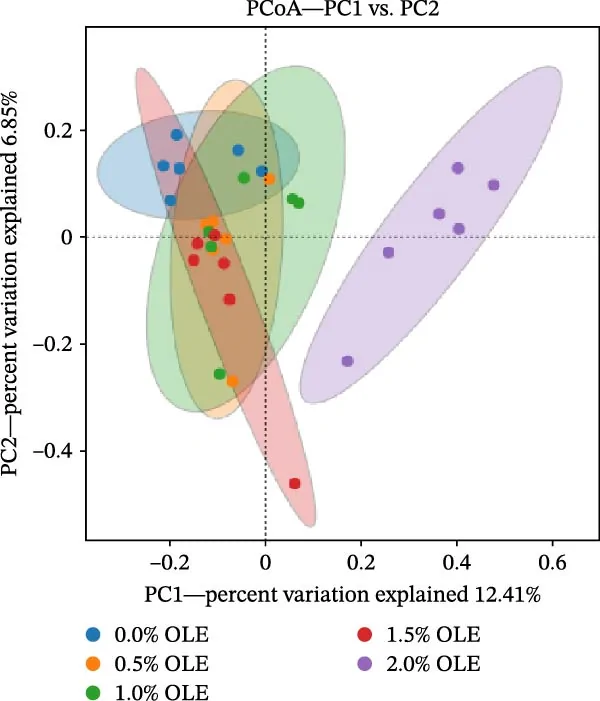
Two‐dimensional principal coordinate analysis (PCoA), using a binary Jaccard distance matrix, of the intestinal bacterial communities of *Trachinotus ovatus* fed high‐fat diets with different levels of olive leaf extract (OLE) for 8 weeks.

Alpha diversity analysis of intestinal flora showed that the coverage of all samples exceeded 98% with no significant differences among the dietary groups (*p* > 0.05) (Table 6). Orthogonal polynomial contrast analysis revealed significant linear and quadratic trends in Chao1, ACE, Simpson, and Shannon as the level of dietary OLE increased (*p* < 0.05). Although the Chao1 index was more variable, it was significantly lowest in *T. ovatus* fed the diet with 2.0% OLE (*p* < 0.05). Compared to fish fed HFD without the addition of OLE, the ACE index tended to reduce with dietary supplementation with OLE and was lowest with the highest level of supplementation of 2.0% (*p* < 0.05). On the contrary, the Shannon and Simpson indices both increased significantly in *T. ovatus* fed OLE‐supplemented diets compared to fish fed the unsupplemented diet, with the highest levels obtained in *T. ovatus* fed diets with 0.5%–1.0% OLE. Principal coordinate analysis (PCoA) of intestinal microbiota showed that there were clear differences in microbiota clustering between *T. ovatus* fed diets with and without OLE, but especially between fish fed the diet supplemented with 2.0% OLE and the other dietary groups (Figure 6).

**Table 6:** Alpha diversity index of intestinal microbiota of *Trachinotus ovatus* fed high‐fat diets with different levels of olive leaf extract (OLE) for 8 weeks^1^.

Parameters	Dietary OLE level (%)					Pooled SEM	*p* value		
	0.0	0.5	1.0	1.5	2.0		ANOVA	Linear	Quadratic
Feature	395.00^ab^	598.00^a^	549.50^a^	647.33^a^	226.25^b^	67.010	0.001	0.187	<0.001
ACE	829.91^a^	578.40^ab^	517.58^bc^	672.65^ab^	273.05^c^	69.207	0.001	<0.001	<0.001
Chao1	659.26^ab^	690.57^ab^	580.12^ab^	756.05^a^	309.31^b^	49.456	<0.001	0.001	0.017
Simpson	0.70^b^	0.97^a^	0.96^a^	0.93^a^	0.89^a^	0.030	<0.001	0.020	<0.001
Shannon	3.48^c^	7.11^a^	6.93^a^	6.41^ab^	5.48^b^	0.350	<0.001	0.006	<0.001
Coverage	0.98	0.99	0.99	0.98	0.99	0.004	0.226	0.144	0.865

*Note:* Means in the same row with different superscript letters are significantly different at *p* < 0.05 as determined by one‐way ANOVA. The linear and quadratic effects of OLE levels were assessed using orthogonal polynomial contrasts with a significance threshold of *p* < 0.05. Feature, different taxonomic units in the microbial community; ACE, abundance‐based coverage estimator; Chao1, species richness estimator; Simpson, relative abundance estimator; Shannon, richness and evenness estimator; coverage, the coverage rate of each sample library.

^1^Values are the means of six replicates.

**Figure 7:**
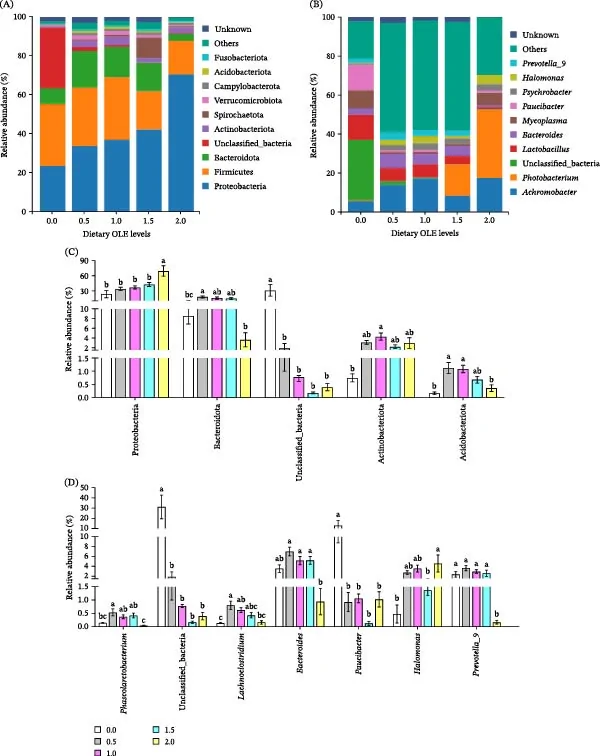
Composition and abundance of bacteria in intestine of *Trachinotus ovatus* fed high‐fat diets with different levels of olive leaf extract (OLE) at the phylum (A, C) and genus (B, D) levels. The top 10 most abundant (based on relative abundance) bacterial phyla and genera are shown, with all other identified phyla and genera combined and assigned as “Others.” Values are presented as means ± SEM, *n* = 6. Bars for each phylum or genus in subfigures (C) and (D) with different superscript lowercase letters are significantly different (*p* < 0.05) as determined by one‐way ANOVA.

At the phylum level, the predominant phyla in overall relative abundance in fish fed HFD without OLE were Proteobacteria, Firmicutes, and Bacteroidetes, with unclassified bacteria also being a major group (Figure 7A). The abundance of Proteobacteria and Actinobacteriota increased progressively with increasing dietary supplementation of dietary OLE, while the abundance of Bacteriodetes and Acidobacteriota showed a trend of first increasing and then decreasing as dietary OLE increased (Figure 7C). At the genus level, the top groups in overall relative abundance in fish fed the control HFD diet without OLE were *Lactobacillus*, *Paucibacter*, and *Mycoplasma*, as well as unclassified bacteria (Figure 7B). Unclassified bacteria and *Paucibacter* tended to decrease in abundance with dietary supplementation with OLE, while *Phascolarctobacterium*, *Lachnoclostridium*, and *Bacteroides* increased and then decreased in abundance as dietary OLE increased (Figure 7D).

## 4. Discussion

### 4.1. OLE Improved Growth Performance and Reduced Lipid Accumulation in *T. ovatus* Fed HFD

Plant extracts have a range of potential beneficial applications, including growth promotion and appetite stimulation in cultured fish [28] and could represent an acceptable and environmentally friendly strategy as feed additives in fish culture in the era of the green economy [29]. In the current study, OLE displayed similar beneficial effects, with WG, SGR, and SR all increased in *T. ovatus* fed HFD supplemented with 0.5% and 1.0% OLE. However, it is important to note that the addition of excessive levels of additives can have negative impacts on the growth and health performance of fish [30]. This was apparent in the current study, where concentrations of OLE above 1.0% had negative effects on the growth of *T. ovatus*, which suggested that dietary supplementation of 1.0% OLE was the optimum level for promoting the growth of *T. ovatus* fed HFD. Similarly, it was reported previously that 200 mg/kg of OLE had a positive effect on the growth of common carp (*Cyprinus carpio*), while higher concentrations showed no beneficial effects [15]. However, other studies reported no significant differences in the growth of rainbow trout (*Oncorhynchus mykiss*) [31] and common carp [32] fed diets with various levels of OLE. The reasons for the differential impacts of OLE on fish growth in different studies may be due to differences in species and biology (age and developmental stage) of the fish studied, the OLE product used (geographical origin, extraction process, and contents/composition of active substances), diet formulations (OLE concentration, lipid content [HFD or not], and other ingredients), or the experimental conditions of the trials.

Dietary lipid level usually impacts whole‐body lipid contents of fish, and excessively high lipid contents in feed are accompanied generally by increased lipid accumulation in tissues including liver, muscle, and abdominal viscera [33]. In the current study, we utilized a lipid level of ~16.0% in the feed to establish an HFD model in *T. ovatus* and found that OLE supplementation of that diet reduced the lipid content of the liver (with significant linear and/or quadratic trends) and whole body. This suggested that dietary OLE can reduce lipid accumulation in tissues of HFD‐fed *T. ovatus*, which was supported by the reduction in both lipid droplet content and size in the liver. These data were consistent with a previous mammalian study that reported that oral administration of 16 mg/kg of oleuropein‐ and hydroxytrosol‐rich OLE improved dyslipidemia induced by HFD in rats [34]. Furthermore, TG and TC concentrations in serum were decreased in *T. ovatus* fed HFD supplemented with OLE, further confirming the impacts of OLE on the regulation of lipid metabolism. The reduced serum TC in *T. ovatus* fed HFD supplemented with 1.0% OLE was associated with decreased serum LDLC but increased HDLC concentrations, reflecting transport of excess cholesterol from peripheral tissues to the liver for metabolism or excretion, accompanied by reduced transport of cholesterol from the liver to peripheral tissues [35].

In general, the accumulation of excess lipid in fish tissues can increase the rate of lipid oxidation that, in turn, can induce the production of reactive oxygen species (ROS) and impact antioxidant capacity [36]. Previous studies showed that oleuropein can increase antioxidant enzyme activities in rats fed HFD [37] and protect human erythrocytes and HeLa cells against oxidative damage [38, 39]. In the current study, supplementation of HFD with OLE increased SOD activity and TAOC and decreased the concentration of MDA in serum, with the optimum effects obtained with 1.0% OLE. This indicated that the antioxidant defense system was increased effectively and oxidative damage reduced by dietary OLE in *T. ovatus* fed HFD. In addition, dietary inclusion of 0.5%–1.0% of OLE decreased activities of AST and ALT in the serum of fish fed HFD, suggesting that an appropriate level of dietary OLE could have an important function in preventing or alleviating liver damage induced by long‐term HFD.

To further explore the impacts of OLE on the liver lipid metabolism, mRNA expression levels of transcription factors and genes related to lipid metabolism were determined. Srebp1 is a transcription factor playing a crucial role in lipogenesis through modulating the expression of adipogenic genes such as *fas*, *acc*, and *dgat1* [40]. In the current study, expression levels of *srebp1*, *acc*, *fas*, and *dgat1* were all reduced in the liver of fish fed HFD supplemented with OLE, particularly at inclusion levels of 0.5% and 1.0%, suggesting that OLE could reduce lipid deposition in the liver by decreasing the rate of lipid synthesis. This was consistent with earlier studies that demonstrated that hydroxytyrosol (a significant component of OLE) mitigated the HFD‐induced liver expression of lipogenesis‐related genes (*SREBP1*, *ACC*, *FAS*, and *SCD1*) in rat [41]. In addition, olive polyphenols were found to have positive effects on lipid metabolism as an antilipogenic agent in medaka (*Oryzias latipes*) [42]. Another important mechanism to reduce lipid deposition is increased lipolysis and fatty acid oxidation. Hsl is a key enzyme in adipocytes, catalyzing TG hydrolysis and providing free fatty acids and glycerol in a hormone‐controlled lipolytic process [43]. In the current study, *hsl* expression in the liver was increased by OLE supplementation, which suggested that OLE may also increase lipolysis in fish fed HFD. Furthermore, pparα is an important transcription factor for mitochondrial β‐oxidation of fatty acids, in which Cpt1 is a pivotal enzyme in long‐chain fatty acid transport into the matrix of mitochondria for oxidation [44]. The transcription of fatty acid transporter genes, *cd36* and *fabp1*, is also regulated by positive feedback of pparα, resulting in increased cellular uptake of fatty acids, which are then transported to mitochondria for β‐oxidation [45]. The current study showed up‐regulated levels of expression of *pparα*, *cpt1*, *hsl*, *cd36*, and *fabp1* in *T. ovatus* fed HFD supplemented with OLE, further suggesting that OLE could increase lipid catabolism in fish liver. The main component of the OLE used in the present study was oleuropein (40%), which has been shown to be an agonist of pparα in rats, playing a role in regulating blood lipids [46, 47]. Thus, it appears that pparα is likely a key factor in the OLE in the regulation of lipid metabolism, but this requires further research. The analysis of liver fatty acid composition revealed that the addition of 0.5%–1.5% OLE to HFD may enhance the ability of *T. ovatus* to utilize C_18_ PUFA, particularly 18:2n–6, whose major fate was indicated to be β‐oxidation for energy production [48], thereby saving LC‐PUFA. Therefore, the impacts of OLE on lipid metabolism in the liver of *T. ovatus* fed HFD may involve the following metabolic mechanisms: (1) reducing lipid synthesis by downregulating the expression of lipogenesis‐related genes and (2) reducing lipid deposition by upregulating the expression of genes related to lipolysis and lipid transport and enhancing β‐oxidation of C_18_ PUFA. However, the detailed mechanisms underpinning the effects that dietary OLE has on the lipid metabolism of fish require further study.

### 4.2. OLE Improved Intestinal Damage and Altered Intestinal Digestive Capacity and Microbiota Composition in *T. ovatus* Fed HFD

As the primary organ for digestion and absorption of nutrients, it is crucial that intestinal functions and morphological integrity are maintained [49]. Well‐developed intestinal folds are conducive to the absorption of nutrients [50] and, in the current study, the height of intestinal folds was increased when 0.5% and 1.0% OLE was supplemented to the diet, which indicated that OLE contributed to a well‐developed intestinal villi area. Supplementation of HFD with 1.0% OLE also increased intestinal wall thickness, which suggested that appropriate dietary OLE did not damage the intestinal mucosa and, rather, was beneficial in maintaining the integrity of the intestinal tissue. In the intestine, goblet cells not only produce mucus that can protect various site‐specific functions but are also involved in immune responses [51]. Previously, we showed that, compared to a standard lipid diet, HFD (18% lipid) triggered intestinal inflammatory responses as evidenced by increased expression of proinflammatory factors *il-8* and *tnfα* in the intestine of *T. ovatus* fed HFD [20]. The current study showed that dietary supplementation with OLE reduced expression levels of proinflammatory cytokines *il-1*, *il-8*, and *tnfα*, and increased goblet cell number per fold in *T. ovatus* fed HFD, which together suggested that OLE can possibly ameliorate HFD‐induced inflammatory responses and support normal protection and lubrication of intestinal mucosa. Moreover, dietary OLE also enhanced intestinal barrier function, as evidenced by upregulation of intestinal barrier function‐related genes *cdh1* and *ocln* in the intestine of *T. ovatus* fed HFD supplemented with 1.0%–1.5% of OLE. This was consistent with the reported effects of OLE on improving intestinal permeability and intestinal barrier function [52]. Furthermore, it was interesting that the addition of OLE at 0.5%–1.5% reduced the activity of intestinal lipase in *T. ovatus* fed HFD. A previous study reported that HFD stimulated the secretion of lipase and induced chronic intestinal inflammation in Nile tilapia [53] and, thus, inhibiting intestinal lipase activity could contribute to the mitigation of inflammation and increased fat deposition in the liver caused by HFD. Therefore, we speculate that one mechanism of action of OLE may be to reduce excess fat deposition in *T. ovatus* fed HFD by lowering the secretion of intestinal lipase and decreasing the digestion and absorption of lipid, thereby breaking the high‐fat intake → excessive fat absorption and deposition → fatty liver pathway. However, further research is required on the mechanism whereby OLE affects the secretion of intestinal lipase.

Intestinal bacteria (microbiota) play important roles in not only the gut barrier but also intestinal function and health [54]. Increased Simpson and Shannon indices, which indicate that intestinal microbiota diversity had increased, were observed with dietary supplementation of OLE in *T. ovatus* fed HFD in the present study. Furthermore, the abundance of bacterial taxa such as *Bacteroides* was increased, while harmful bacterial taxa like *Mycoplasma* were decreased in *T. ovatus* fed diets supplemented with OLE. Studies in rats showed previously that OLE can prevent the reduction of *Bacteroides* caused by HFD [52]. *Bacteroides* are considered to be beneficial bacteria that can reduce lipid accumulation, decrease hyperlipidemia, and prevent hepatic steatohepatitis and liver injury in rats [55]. Other studies reported that *Lactobacillus* can promote the in vivo conversion of oleuropein to hydroxytyrosol [56]. It is noteworthy that ~20% of the bacteria were unclassified in the intestinal microbiota of *T. ovatus* fed the control HFD, and this was reduced as dietary supplementation with OLE increased. The reduction of uncategorized bacteria in fish fed diets containing OLE may be a further reason for the alleviation of intestinal inflammation and damage caused by feeding HFD in the present study. Further identification of the unclassified bacterial species will be required in the future to further reveal possible mechanisms by which OLE alleviated intestinal damage caused by HFD. Overall, the results suggested that OLE supplementation favorably altering the composition of the intestinal microbiota in *T. ovatus* fed HFD, which can possibly supplement the roles of oleuropein in decreasing blood lipids, supporting antioxidation, and improving immunity.

## 5. Conclusion

In conclusion, dietary OLE supplementation promoted growth, reduced lipid accumulation by modulating lipid metabolism, promoted antioxidant and immune capacities, reduced intestinal inflammation responses and intestinal lipase activity, protected intestinal integrity, regulated microbial community structure, and thus improved intestinal health in *T. ovatus* fed HFD. An inclusion level of 1.0% OLE provided demonstrable advantages across multiple physiological parameters in *T. ovatus* fed HFD. Overall, the current findings have contributed to the alleviation of the problem of excessive lipid accumulation in the liver of fish induced by feeding HFD and the understanding of the beneficial role of OLE, which further supports the development and application of plant extracts as feed additives in aquaculture.

## Author Contributions

**Jiaying Xie**: writing – original draft. **Jiajian Shen**: data curation, investigation, software. **Douglas R. Tocher**: writing – review and editing. **Yuanyou Li**: conceptualization, methodology. **Yansen Hao, Zeling Lin, and Han Zhan**: investigation. **Fan Lin**: methodology. **Shuqi Wang**: conceptualization, funding acquisition, methodology. **Cuiying Chen**: conceptualization, funding acquisition, methodology, project administration, writing – review and editing.

## Funding

This study was supported financially by the Special Fund Project of Science and Technology Application in Guangdong Province (Grant STKJ202209037), the Guangdong Province “Hundreds of Millions Project” County‐level Innovation Base (Nan’ao County, Shantou City) (Grant STKJ2024003), the Guangdong Province Rural Science and Technology Commissioner Project (Grant KTP20240854), and the Guangdong Agriculture Research System (Grant 2023KJ150).

## Disclosure

A preprint of this manuscript was made publicly available on Research Square prior to the journal submission [57]. The preprint has not undergone peer review, and this submission represents the first formal peer review request for the work, and there are no substantive differences between the submitted manuscript and the preprint.

## Conflicts of Interest

The authors declare no conflicts of interest.

## Supporting Information

Additional supporting information can be found online in the Supporting Information section.

## Supporting information


**Supporting Information** Table S1: Primer information of target genes used for qPCR in this study. Table S2: The fatty acid composition in liver tissue of pompano fed diets with different OLE levels for 8 weeks.

## Data Availability

All the results of the study presented in the manuscript are available from the corresponding author upon reasonable request.
