# Supplementary material for: Dietary Supplementation With Olive Leaf Extract Alleviated Lipid Accumulation and Improved Growth and Intestinal Flora in Trachinotus ovatus Fed High‐Fat Diet
Source: Aquac Nutr. 2026 Aug 21;2026:8894218. doi: 10.1155/anu/8894218 (PMC13498860; doi:10.1155/anu/8894218)
Supplement: Supplementary file 1 [file ANU-2026-8894218-s001.docx]

**Table S1. Primers of target genes used for qPCR**

| Target gene | Forward primer (5’-3’) | Reverse primer (5’-3’) | Size (bp) |
| --- | --- | --- | --- |
| *acc*^[1]^ | GTTGTCAATCCCAGCCGATC | ATCCACAATGTAGGCCCCAA | 226 |
| *cd36*^[1]^ | CTCTTCATGGAAAGCAGGCC | CAGCTCCGAATACAACAGCC | 154 |
| *cpt1*^[2]^ | CTTTAGCCAAGCCCTTCATC | CACGGTTACCTGTTCCCTCT | 108 |
| *dgat1*^[3]^ | GTGTCTGCCTTCTTCCACGA | TTCCATTCAGGAAGCGACCC | 124 |
| *fas*^[2]^ | GAAGGAGAGGGGGTGGAGTC | GTGTGAAGGTGGAGGGTGTG | 118 |
| *fabp1*^[2]^ | AGTCATTGTCTGGGGAGGG | GTCAAGGCGGTGGTTCA | 198 |
| *hsl*^[1]^ | TCATACCTCCACACCAACCC | GTCTCGCAGTTTCTTGGCAA | 241 |
| *pparα*^[4]^ | AATCTCAGCGTGTCGTCTT | GGAAATGCTTCGGATACTTG | 204 |
| *srebp1*^[1]^ | GAGCCAAGACAGAGGAGTGT | GTCCTCTTGTCTCCCAGCTT | 169 |
| *zo-1*^[4]^ | CGACAAAGAGAAAGGAGAAACG | AGAAGTGGTCAATGAGCACAGATA | 191 |
| *cdh1*^[4]^ | CCCTGCTGGTGTTTGACTAT | GCAGTCGTAATCCTGGTCTC | 95 |
| *ocln*^[5]^ | TACGCCTACAAGACCCGCA | CACCGCTCTCTCTGATAAA | 174 |
| *il-8*^[5]^ | TGCATCACCACGGTGAAAAA | GCATCAGGGTCCAGACAAATC | 140 |
| *tnfα*^[4]^ | GCTCCTCACCCACACCATCA | CCAAAGTAGACCTGCCCAGACT | 237 |
| *il-1*^[4]^ | TGCATCACCACGGTGAAAAA | GCATCAGGGTCCAGACAAATC | 140 |
| *β-actin*^[6]^ | CGCCCGAGTGTGTATGAGAAATG | GTCATGTGGATCAGCAAGCAGGA | 166 |

*acc*, acetyl-CoA carboxylase; *β-actin*, actin beta; *cd36*, CD36 molecule; *cdh1*, cadherin 1; *cpt1*, carnitine palmitoyl transferase-1; *dgat1*, diacylglycerol acyltransferase 1; *fas*, fatty acid synthase; *fabp1*, fatty acid binding protein 1; *hsl*, hormone sensitive lipase; *il-1*, interleukin-1; *il-8*, interleukin-8; *ocln*, occludin; *pparα*, peroxisome proliferator-activated receptor α; *srebp1*, sterol regulatory element binding protein 1; *tnfα,* tumor necrosis factor alpha; *zo-1*, zonula occludens-1.

**Table S2. Fatty acid compositions of liver of golden pompano fed diets with different OLE levels for 8 weeks.^1^**

| Fatty acid | Dietary OLE levels | | | | | Pooled  SEM | *P* value | | |
| --- | --- | --- | --- | --- | --- | --- | --- | --- | --- |
|  | 0.0 | 0.5 | 1.0 | 1.5 | 2.0 |  | ANOVA | Linear | Quadratic |
| 14:0 | 0.52^b^ | 0.84^a^ | 0.79^ab^ | 0.93^a^ | 0.89^a^ | 0.059 | 0.005 | 0.001 | 0.037 |
| 16:0 | 13.76 | 17.22 | 16.89 | 20.30 | 17.26 | 1.785 | 0.230 | 0.105 | 0.196 |
| 18:0 | 17.51 | 17.41 | 17.88 | 15.32 | 15.05 | 1.315 | 0.435 | 0.122 | 0.508 |
| 20:0 | 1.22 | 1.79 | 1.75 | 1.40 | 1.47 | 0.345 | 0.743 | 0.908 | 0.330 |
| 16:1n-9 | 1.61 | 2.17 | 1.62 | 1.72 | 1.49 | 0.299 | 0.563 | 0.487 | 0.422 |
| 18:1n-9 | 12.16^b^ | 14.62^ab^ | 18.17^a^ | 16.50^ab^ | 16.01^ab^ | 1.271 | 0.054 | 0.033 | 0.036 |
| 18:2n-6 | 39.96 | 31.19 | 29.05 | 30.00 | 33.03 | 2.373 | 0.055 | 0.073 | 0.013 |
| 18:3n-3 | 1.85 | 1.59 | 1.45 | 1.18 | 1.51 | 0.334 | 0.721 | 0.326 | 0.425 |
| 20:2n-6 | 4.22 | 4.02 | 3.68 | 3.45 | 3.63 | 0.634 | 0.909 | 0.403 | 0.729 |
| 20:3n-3 | 0.60 | 0.81 | 0.76 | 0.74 | 0.63 | 0.142 | 0.827 | 0.983 | 0.295 |
| 20:3n-6 | 0.47 | 0.43 | 0.65 | 0.41 | 0.36 | 0.201 | 0.867 | 0.711 | 0.525 |
| 20:4n-6 | 0.85 | 0.93 | 1.06 | 0.64 | 0.68 | 0.116 | 0.138 | 0.124 | 0.177 |
| 20:5n-3 | 1.18 | 1.41 | 1.14 | 1.19 | 1.16 | 0.179 | 0.810 | 0.661 | 0.760 |
| 22:5n-6 | 0.29 | 0.39 | 0.40 | 0.47 | 0.37 | 0.051 | 0.260 | 0.175 | 0.116 |
| 22:5n-3 | 0.29 | 0.39 | 0.38 | 0.50 | 0.48 | 0.061 | 0.193 | 0.033 | 0.641 |
| 22:6n-3 | 1.50 | 1.83 | 2.37 | 2.42 | 2.20 | 0.330 | 0.299 | 0.085 | 0.225 |
| SFA | 33.01 | 37.27 | 37.32 | 39.95 | 34.66 | 2.179 | 0.481 | 0.576 | 0.106 |
| MUFA | 13.76 | 16.79 | 19.79 | 18.22 | 17.50 | 1.314 | 0.080 | 0.058 | 0.034 |
| PUFA | 51.21 | 42.99 | 40.94 | 41.00 | 44.05 | 2.596 | 0.097 | 0.075 | 0.030 |
| n-3 PUFA | 5.42 | 6.03 | 6.10 | 6.03 | 5.97 | 0.477 | 0.847 | 0.480 | 0.432 |
| n-6 PUFA | 44.94^a^ | 36.04^ab^ | 33.77^b^ | 34.33^ab^ | 37.39^ab^ | 2.281 | 0.037 | 0.042 | 0.011 |
| LC-PUFA | 9.40 | 10.22 | 10.44 | 9.83 | 9.51 | 0.986 | 0.929 | 0.956 | 0.419 |

^1^Values are the means of three replicates. Means in the same row with different superscript letters are significantly different at *P* < 0.05 as determined by one-way ANOVA. The linear and quadratic effects of OLE levels were assessed using orthogonal polynomial contrasts with a significance threshold of *P* < 0.05. SFA, saturated fatty acids including 14:0, 16:0, 18:0 and 20:0; MUFA, monounsaturated fatty acids including 16:1n-9 and 18:1n-9; PUFA, polyunsaturated fatty acids including 18:2n-6, 20:2n-6, 20:3n-6, 20:4n-6, 18:3n-3, 20:3n-3, 20:5n-3, 22:5n-6, 22:5n-3 and 22:6n-3; LC-PUFA, long-chain polyunsaturated fatty acids including 20:2n-6, 20:3n-6, 20:4n-6, 20:3n-3, 20:5n-3, 22:5n-6, 22:5n-3 and 22:6n-3.

**References**

[1]	Li, M. M., Zhang, M., Ma, Y. C., et al. (2020). Dietary supplementation with n-3 high unsaturated fatty acids decreases serum lipid levels and improves flesh quality in the marine teleost golden pompano *Trachinotus ovatus*. *Aquaculture, 516*, 734632. doi:<https://doi.org/10.1016/j.aquaculture.2019.734632>

[2]	Liu, K., Liu, H. Y., Chi, S. Y., et al. (2018). Effects of different dietary lipid sources on growth performance, body composition and lipid metabolism-related enzymes and genes of juvenile golden pompano, *Trachinotus ovatus*. *Aquac Res, 49*(2), 717-725. doi:<https://doi.org/10.1111/are.13502>

[3]	Zhang, G. R., Xu, J. Z., Chen, F., et al. (2025). Dietary fatty acid composition modulates growth in the marine teleost *Trachinotus ovatus* by regulating the hepatic triglyceride to (phosphatidylcholine + phosphatidylethanolamine) ratio via the Ampk/Srebp signaling pathway. *Aquaculture, 607*, 742660. doi:<https://doi.org/10.1016/j.aquaculture.2025.742660>

[4]	You, C. H., Chen, B. J., Wang, M., et al. (2019). Effects of dietary lipid sources on the intestinal microbiome and health of golden pompano (*Trachinotus ovatus*). *Fish Shellfish Immun, 89*, 187-197. doi:<https://doi.org/10.1016/j.fsi.2019.03.060>

[5]	Tan, X. H., Sun, Z. Z., Zhou, C. P., et al. (2018). Effects of dietary dandelion extract on intestinal morphology, antioxidant status, immune function and physical barrier function of juvenile golden pompano *Trachinotus ovatus*. *Fish & Shellfish Immunology, 73*, 197-206. doi:10.1016/j.fsi.2017.12.020

[6]	Tan, X. H., Sun, Z. Z., Huang, Z., et al. (2017). Effects of dietary hawthorn extract on growth performance, immune responses, growth- and immune-related genes expression of juvenile golden pompano (*Trachinotus ovatus*) and its susceptibility to Vibrio harveyi infection. *Fish Shellfish Immun, 70*, 656-664. doi:<https://doi.org/10.1016/j.fsi.2017.09.041>
